# Effects of Different Soybean and Coconut Oil Additions on the Physicochemical and Sensory Properties of Soy Protein–Wheat Protein Mixture Subjected to High-Moisture Extrusion

**DOI:** 10.3390/foods13142263

**Published:** 2024-07-18

**Authors:** Wentao Zhang, Bowen Hui, Xuejie Li, Zengwang Guo, Jian Ma, Jian Li

**Affiliations:** 1 School of Food and Health, Beijing Technology and Business University, Beijing 100048, China; 20221107@btbu.edu.cn (W.Z.); 15511576581@163.com (B.H.); 2150021007@st.btbu.edu.cn (X.L.); 2350201007@st.btbu.edu.cn (J.M.); 2 Key Laboratory of Green and Low-Carbon Processing Technology for Plant-Based Food of China National Light Industry Council, Beijing 100048, China; 3 Department of Environmental Protection and Biopharmaceutical, Beijing Industrial Technician College, Beijing 100048, China; 4 College of Food Science, Northeast Agricultural University, Harbin 150030, China; gzwname@neau.edu.cn

**Keywords:** protein mixture, high-moisture extrusion, soybean oil, coconut oil, physiochemical properties

## Abstract

A protein mixture was prepared using a blend of soybean protein isolate, soybean protein concentrate, and wheat protein through high-moisture extrusion. This study investigated the effects of soybean oil/coconut oil additions (2%, 5%, and 8%) on the physiochemical properties of a soy protein–wheat protein mixture subjected to high-moisture extrusion. The protein extrudates underwent assessment for textural properties, fiber degree, sensory evaluation, microstructure, protein solubility, and protein secondary structure. The findings indicated that plant oils significantly reduced the hardness, springiness, and chewiness of the extrudates, and 5% plant oil significantly increased the fiber degree of the extrudates. In addition, the highest fiber degree and sensory evaluation score were achieved with 5% coconut oil. Observation of the macro- and microstructure indicated that the presence of unsaturated fatty acids in soybean oil did not benefit the improvement of the fibrous structure of protein extrudates during high-moisture extrusion processing. SDS-PAGE and FTIR results revealed that coconut oil, rich in saturated fatty acids, caused the clustering of medium- and low-molecular-weight subunits in texturized protein. Additionally, coconut oil elevated the ratio of 11S protein subunits containing sulfur-based amino acids and facilitated a shift from *β*-turn to *β*-sheet. The inclusion of plant oils increased the development of hydrogen and disulfide bonds, resulting in a denser, fibrous structure. DSC demonstrated that plant oils reduced the thermal stability of the texturized proteins but enhanced the order of protein structure.

## 1. Introduction

The rising global population is leading to an increased need for meat as a source of protein. This pattern will cause animal farming to expand, resulting in substantial adverse effects on land, water resources, and environmental alterations [1]. An increasing number of individuals are becoming aware of the environmental, moral, and health concerns linked to consuming animal products, resulting in a shift towards vegetarian or vegan diets. Plant proteins can be texturized through high-moisture extrusion to simulate the meat-like texture and sensory perception of animal meat such as beef, chicken, and pork [2]. However, despite being a sustainable protein source that meets consumer standards, plant-based meat counterparts are still not completely comparable to their animal-based counterparts.

Plant proteins, including soy protein, wheat gluten, and peanut protein, are the most frequently utilized ingredients in protein extrudates. Typically, use of various kinds of plant protein mixture could significantly improve the quality of extrudates [3]. High-moisture extrusion is a relatively new technology for producing protein extrudates with fibrous structure [4]. The extrusion process includes feeding, mixing, cooking, and cooling zone operations. Initially, raw materials and water are mixed in the feeding zone. Next, the blend is conveyed to the mixing zone and thoroughly mixed to an even consistency. Protein physicochemical properties in the cooking zone experience notable alterations as a result of the combined impact of the synergistic effects of elevated temperatures, pressures, and shear forces. Afterward, within the cooling zone, the molten substances experience variations in pressure, shear stress, and temperature, leading to the reorganization of the protein molecules. During these operations, the protein structure undergoes unfolding, association, aggregation, and cross-linking, which facilitate the protein–protein interactions, thus forming a fibrous structure with a specific orientation [5]. Currently, the taste of the high-moisture extrusion soy protein did not completely satisfy consumers in terms of succulent texture, perceived softness, and desired meaty taste. To further improve the sensory properties of high-moisture extrusion soy protein, adding lipids during the extrusion process should be necessary and helpful.

Lipids (plant oil or fat) were reported to contribute to improving the tenderness and juiciness of protein extrudates and have drawn increased attention recently [6]. Hence, plant-based meat substitutes often use sunflower, canola, corn, sesame, coconut, or palm oils to enhance texture and taste. Meanwhile, previous studies have suggested that lipids could act as emulsifiers, plasticizers, and lubricants in extrusion processing, potentially impacting both the extrusion process and the formation of fibrous structures [7]. It has been shown that the effect of lipids on the performance of extrudates depends on their addition amount, process parameters, and type of lipids. The study by Li et al. [8] suggested that adding (lard, butter, and sunflower oil) to duck egg white was shown to promote the exposure of hydrophobic regions, hydrophilic polar groups, and sulfhydryl groups within the duck egg white gel molecules, thereby enhancing the hydrophobic interactions and cross-linking via disulfide bond formation (between gel proteins, between interfacial film and gel proteins) within the gels. Devi et al. [9] studied the impact of six fats and oils with differing saturated fatty acid content on the quality of cookie dough and cookies and found that an appropriate addition of fats and oils facilitated the formation of a layered structure in the dough, while fats and oils high in saturated fatty acids adversely affected the hardness and springiness of the cookies. The study by Kendler et al. [10] found that when 4% oil was added, wheat gluten lost its ability to create fibrous structures and experienced a significant decrease in Young’s modulus. However, it is important to note that the plant oils utilized in creating plant protein meat alternatives had significantly different fatty acid compositions. Saturated and unsaturated fats showed different melting temperature, emulsification characteristics, and protein interactions, impacting how the protein matrix flows in the extruder and the formation of anisotropic structures. Yet, there remains a lack of specific information regarding the impact of plant oils with different fatty acid compositions on the formation of fibrous structures in plant-protein extrudates during the high-moisture extrusion process. 

Soybean oils are rich in unsaturated fatty acids, whereas coconut oil is abundant in saturated fatty acids. Soybean oil and coconut oil differ significantly in fatty acid composition. Therefore, the aim of this study was to understand the influence of soybean oil and coconut oil with different fatty acid compositions on high-moisture extrudates based on soybean protein isolate (SPI) on a molecular level. Additionally, the impact of plant oils on the quality of the extrudate, such as its microstructure (SEM), color (L*, a* and b*), texture (hardness, springiness, and chewiness), and fiber degree, as well as sensory properties, molecular weight distribution of protein 7S and 11S (SDS-PAGE), secondary structure (FTIR), and solubility were assessed to offer valuable insights for utilizing plant oils in creating plant-protein-based meat alternatives.

## 2. Materials and Methods

### 2.1. Materials

SPI (protein content was 90.5%) was provided by Yuwang Ecological Food Industry Co., Ltd. (Dezhou, China). Soybean protein concentrate (SPC, protein content was 69%) powder was obtained from Wonderful Industrial Group Co., Ltd. (Dongying, China). Wheat protein (WP, protein content was 85.3%) powder was purchased from Huafeng Powder Industry Co., Ltd. (Xinxiang, China). The soybean oil and coconut oil were purchased from Yihai Kerry Foodstuffs Industries Co., Ltd. (Tianjin, China). All other reagents used in this study, such as 2-mercaptoethano, urea, and sodium dodecyl sulfate (SDS), were analytically pure reagents.

### 2.2. Preparation of High-Moisture Extruded Protein

Initially, SPI, SPC, and WP dry powder blends were created in a 3:3:4 ratio using a powder mixer. Next, the dry powder mixtures were combined with soybean oil and coconut oil, individually. The additive amount of plant oil added was set at 2%, 5%, and 8% (*w*/*w*, dry basis). The dry powder mixtures without plant oil were used as the control group. Extrusion trials were performed using a co-rotating twin-screw extruder (AHT36, Arrow Machinery Co., Ltd., Jinan, China) with a screw diameter of 19 mm and length-to-diameter (L/D) ratio of 32. A rectangular cooling die [70 mm (width) × 6 mm (height) × 1000 mm (length)] was coupled to the extruder through a die adapter. The extruder barrel was segmented into five temperature-controlled zones that were electrically heated and cooled with water-cooling inserts inside the barrel. The temperature in the cooling die was regulated by tap water functioning as a cooling medium. SPI, SPC, and WP dry powder blends were combined at a steady flow of 10 kg/h and introduced into the extruder, with deionized water being added at a constant rate of 15 kg/h, leading to a moisture content of 60%. Tests were carried out with a constant screw velocity of 480 rpm. The barrel temperatures were set at 30 °C, 70 °C, 110 °C, 130 °C, and 170 °C in zones 1 to 5. The two temperature-controlled zones of the cooling die were maintained at 60 and 40 °C. Extrudate samples were collected from the cooling die outlet after reaching a steady state. The samples were immediately frozen and stored at −20 °C.

### 2.3. Color Evaluation

The extrudates’ color, such as lightness (L*), redness (a*), and yellowness (b*), was assessed with a colorimeter (CR-400, Konica Minolta, Tokyo, Japan). The process was repeated three times for each sample.

### 2.4. Textural Analysis and Fiber Degree

Texture analysis of the extrudate was analyzed with a TMS-Pro texture analyzer (Food Technology Corporation, Sterling, VA, USA). The extruded samples were cut into 15 × 15 × 5 mm^3^ cubes and tested under the ‘Texture Profile Analysis (TPA)’ pattern for hardness, springiness, and chewiness analysis. Each sample was compressed to 50% of its original thickness at a speed of 1.5 mm/s for 10 s using a TMS-2.5 mm steel probe. To assess fiber degree of structure formation, a knife blade probe was used to cut at a rate of 1 mm/s transverse and lengthwise to 50% of its initial thickness. The fiber degree was defined as the ratio of the transverse shear force (Fv) to the longitudinal shear force (Fp) [11]. The transverse shear force (Fv) is defined as the force received by the cut perpendicular to the direction of extrusion, while the longitudinal shear force (Fp) is defined as the force received by the cut parallel to the direction of extrusion. The process was repeated three times for each sample.

### 2.5. Sensory Evaluation

All panelists gave their informed consent to participate before the sensory trial. Beijing Technology and Business University ethics committee reviewed the protocol for performing the sensory evaluation. It follows the Helsinki declaration. The approval protocol number is No. 57 of 2024. The sensory evaluation was carried out by a panel of ten trained assessors (five females and five males, Beijing, China) with an average age of 30 years, who had previously received professional training in sensory evaluation of food in the laboratory. Five indicators were involved in the sensory evaluation, mainly including color, appearance, texture, flavor, and mouthfeel. For the sensory evaluation, each sample was placed in a clear plastic cup and randomly numbered using a three-digit code. The assessors had to rinse their mouths with warm water after each sample test. The scoring criteria detailed in Table 1 was based on the report by Xiao et al. (2022) [12]. The overall sensory score was calculated using the following formula:

Sensory score=∑score of each attribute × coefficients of correspondence

### 2.6. Macroscopic and Microscopic Morphology

To observe the macrostructure of the samples, the samples were freeze-dried and photographed to visualize their internal anisotropic structures from front, top, and side views.

In order to examine the microstructure of the specimens, the microstructure of the specimens was examined and imaged with a scanning electron microscope (S-3400n, Hitachi Ltd., Tokyo, Japan) at magnifications of 300×, 500×, and 1500×. The method of sample pretreatment referred to the research of Guo et al. [11].

### 2.7. SDS-Polyacrylamide Gel Electrophoresis (SDS-PAGE)

The SDS-PAGE followed the methodology described by Chen et al. [13], albeit with specific adjustments. Specifically, 2 mg of sample powder was ground and sieved and then dissolved in buffer. Next, the specimen was placed in a water bath at boiling temperature for 5 min before being allowed to cool to room temperature. Next, the sample solutions were added to the electrophoresis lane in a 5 μL volume. Following the completion of electrophoresis, the gel was removed and stained for one hour prior to decolorization.

### 2.8. Fourier-Transform Infrared Spectroscopy (FTIR)

The freeze-dried powder sample was mixed with potassium bromide in a mass ratio of 1:100 before being compacted into thin slices. Subsequently, the FTIR (8400S, Shimadzu, Kyoto, Japan) was used to take measurements within the wavelength range of 1000–3600 cm^−1^. PeakFit 4.12 was utilized to conduct the analysis.

### 2.9. Protein Solubility and Chemical Interactions Analysis

To measure chemical interactions of samples, preparation of seven reagents was carried out using 0.05 M phosphate buffer, which included 0.035 mol/L phosphate buffer (P), P + 8 mol/L urea (A), P + 0.1 mol/L 2-mercaptoethanol, 2-ME (B), P + 1.5% SDS (C), P + 8 mol/L urea + 0.1 mol/L 2-ME (D), P + 8 mol/L urea + 1.5% SDS (E), and P + 8 mol/L urea + 1.5% SDS + 0.1 mol/L 2-ME (F). After combining 100 mg of sifted powder with 5.0 mL of the specified reagents for 120 min, the content of protein in the supernatant was measured using the Coomassie Brilliant Blue technique by a Bradford kit (Solarbio Science & Technology Co., Ltd., Beijing, China). The protein content disparity between A and P, B and P, and C and P was denoted as the levels of hydrogen bond concentration, disulfide bond concentration, and hydrophobic interactions concentration, respectively [14].

### 2.10. Differential Scanning Calorimetry (DSC)

According to the previous approach by Grabowska et al. [15], the thermal properties of protein extrudates were determined. The powder samples were placed in a pan made of aluminum and then sealed. The temperature range was 30–220 °C at a rate of 10 °C/min. 

### 2.11. Statistical Analysis

The results measurements were repeated three times, and they were presented as mean ± SD. Duncan’s multiple range test and the one-way analysis of variance were performed by SPSS 18.0, plotted and date analyzed using Origin 2021.

## 3. Results and Discussion

### 3.1. Textural Properties and Fiber Degree

**Figure 1:**
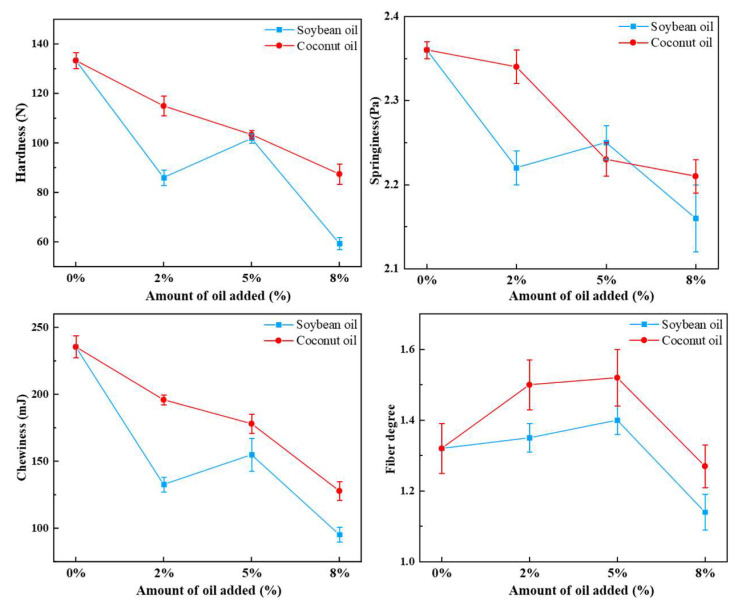
Effect of plant oil on textural properties and fiber degree of the texturized protein.

Textural characteristics such as hardness, springiness, and chewiness could reflect the processing properties of texturized soybean protein products. The impact of plant oils on the textural properties and fiber degree of texturized soybean protein products is shown in Figure 1. Compared with the control sample, the plant oils significantly reduced these textural characteristics (hardness, springiness, and chewiness). As the soybean oil content increased, the hardness, springiness, and chewiness of the texturized protein products showed the same trend, which first increased and then decreased. These textural characteristics reached the peaks at a 5% soybean oil content, which were 101.82 N, 2.25 Pa, and 154.86 mJ, respectively. In contrast, the addition of coconut oil resulted in a continuous decrease in these textural characteristics. The hardness, springiness, and chewiness of the texturized protein products reached minimum values at 8% coconut oil content, which were 87.44 N, 2.21 Pa, and 127.89 mJ, respectively. The possible reason was that during the extrusion process, the lubricating effect of oil broke the protein network, and the aggregation of lipid droplets in the protein network could provide flexible structural domains, leading to a decrease in the hardness of textured protein products [16]. The findings that we obtained were consistent with the research by Wang et al. [17], who discovered that when the ratio of oil/water increases, hardness and chewiness could decrease in SPI extrudates.

The fiber degree of texturized soybean protein products (Fv/Fp) is a sign of fiber structure formation, when Fv/Fp > 1.0, indicating predominantly longitudinal fibers [18]. Figure 1 shows that all samples had prominent longitudinal fibers with Fv/Fp > 1.0. Moreover, when the oil content was increased, the fiber degree of texturized protein products increased first and then decreased. Specifically, 5% coconut oil significantly enhanced the fiber degree of texturized protein products to 1.52, whereas the addition of soybean oil did not significantly affect the fiber degree. Adding a small amount of oil might serve as a lubricant, reducing the shear and friction inside the cooling die, which helped improve the tenderness and fiber degree of the texturized soybean protein products. However, excessive oil content may lead to interactions between fatty acids and hydrophobic groups on the surface of proteins, inhibiting protein cross-linking, which resulted in a loose structure [19].

### 3.2. Color of the Extrudates

**Figure 2:**
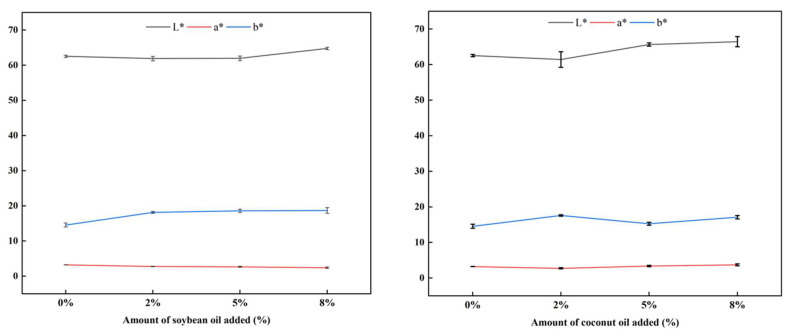
Effect of plant oil on color of the texturized protein.

Product color is a key indicator that determines consumer acceptance. Color values including L*, a*, and b* could not only be used to quantify the visual appearance of product but also indirectly reflect the degree of chemical reactions [20]. Figure 2 presents the effect of plant oils on the color values of the extrudates. With the increase in the addition of soybean oil, the lightness (L*) of the extrudates first decreased slightly and then increased. But for coconut oil, when the content of coconut oil increased, the L* of the extrudates gradually increased, and the L* of coconut oil groups was higher than the soybean oil groups. This was mainly due to the differences in the contributions of different plant oils to light reflection. Furthermore, the presence of soy oil produces less red and more yellow, which means that unsaturated fatty acids might prevent the formation of colored substances. As the amount of coconut oil increased, the a* value first decreased and then increased, and the b* value was higher than the control group, resulting in a brighter overall color of the extrudates. This indicated that the addition of saturated fatty acids was beneficial for producing pigments that were close to meat color. From this, we infer that soybean oil reduced the overall color of the extrudates. The low-molecular-weight carbonyl compounds produced by the decomposition of unsaturated fatty acids in soybean oil could condense with free amino groups to form Schiff bases, further generating brown macromolecular polymers, thereby darkening the color of the product [21].

### 3.3. Sensory Evaluation

**Figure 3:**
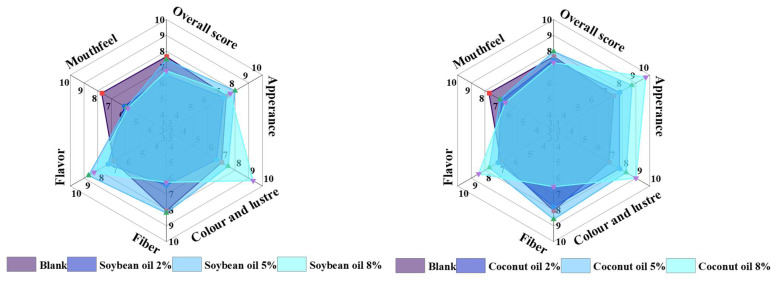
Effect of plant oil on the sensory evaluation of the texturized protein.

Figure 3 shows a radar chart illustrating the sensory evaluation results of the texturized protein products of the control, 5% soybean oil groups, and 5% coconut oil groups. The coconut oil groups outperformed the soybean oil groups in appearance, color, and texture, while the soybean oil groups scored higher than the coconut oil groups in flavor. Although the texturized protein prepared by both oil groups had a slightly lower mouthfeel than the control group, adding oil improved the sensory properties of texturized protein in general.

**Figure 4:**
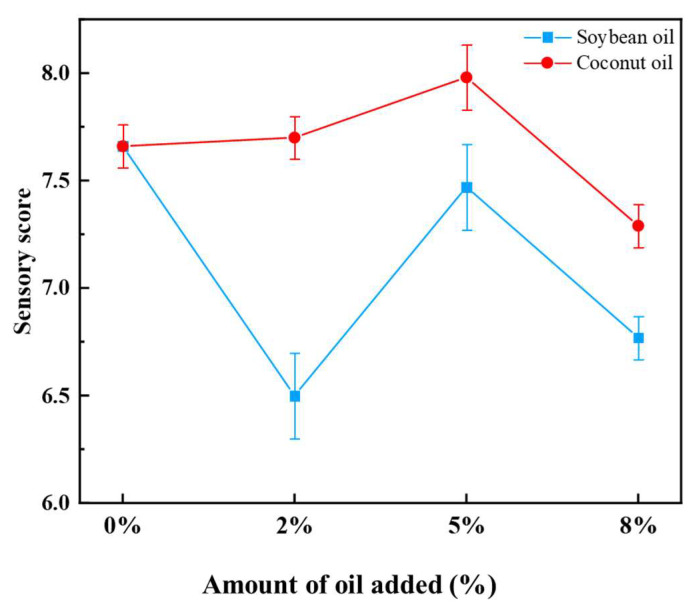
Effect of plant oil on the sensory weighted score of the texturized protein.

Figure 4 illustrates the impact of plant oils on the sensory evaluation scores of the texturized protein products. As the oil content increased, a marked divergence in sensory scores was observed between the two oils. Products containing soybean oil generally exhibited a decline in sensory evaluation scores. The scores of the texturized protein products with 2% and 5% coconut oil were slightly higher than those of the control sample, with the lowest score recorded at an 8% oil content. The overall weighted scores calculated using the formula in Section 2.5 were in the order of 5% coconut oil > control > 5% soybean oil. This suggested that appropriate addition of oils high in saturated fatty acids could improve the sensory properties of texturized soybean protein products.

### 3.4. Macroscopic and Microscopic Morphology

**Figure 5:**
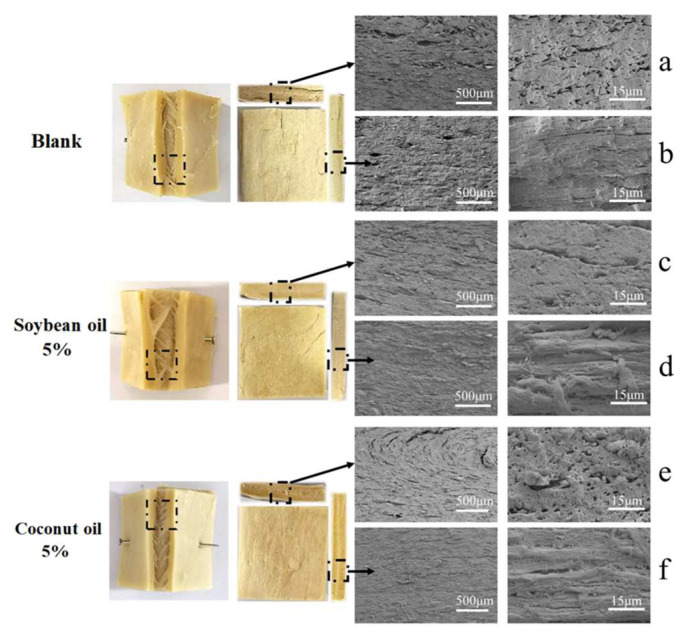
Microstructure of the texturized protein with different plant oils, obtained by SEM. (**a**,**c**,**e**): Transversal sections of soybean protein extrudates at 30× and 1500× magnification. (**b**,**d**,**f**): Longitudinal cross-sections of soybean protein extrusions at 30× and 1500× magnification. The black frames in each picture represent the areas where the fibrous structure is most evident.

To further investigate the macroscopic and microscopic morphology, we compared the product structures of the control group and the 5% soybean oil groups and 5% coconut oil groups, using magnifications of 30× and 1500×. As shown in Figure 5, at the macro-level, the texturized protein with soybean oil had a rough and dark surface and lacked a clear arrangement of fibers in the cross-section. In contrast, the texturized protein with coconut oil displayed uniform fibers, a smooth surface, a light color, and a uniform fiber arrangement. Previous research indicated that during the extrusion process, the formation of complexes between oils and proteins could promote protein molecules to cause cross-linking and rearrangement, thus enhancing the effectiveness of the extrusion process [22]. The cross-section images of samples are shown in Figure 5a,c,e. In the control sample, the pore structure of extrudates was disordered and loose. A looser and more porous structure can be seen on the cross-section of texturized protein with soybean oil, while there were denser structures in the extrudates of coconut oil. The longitudinal section images of samples are shown in Figure 5b,d,f, and the protein extrudates in the control group showed a less coherent structure and were losing layered texture. The layered structure of soybean oil protein extrudates was obvious, while protein extrudates with coconut oil showed a more compact and homogeneous layered texture. Chen et al. [13] found in their research that adding a certain amount of oil rich in saturated fatty acids was beneficial for the formation of dense, meat-like fibrous structures in the extruded material. This result was consistent with our results.

### 3.5. SDS-PAGE

**Figure 6:**
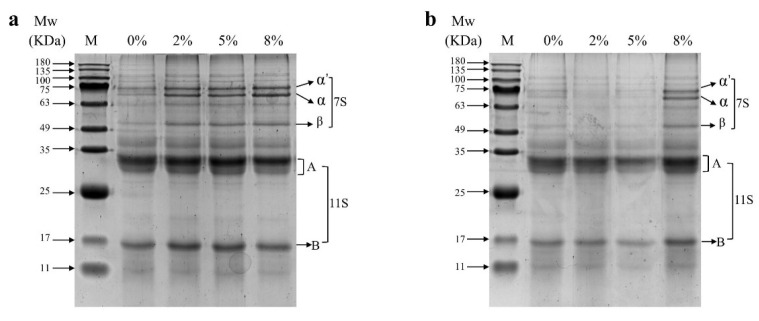
SDS-PAGE patterns of soybean oil (**a**) and coconut oil of protein extrudates (**b**).

Soybean contains two main proteins, which are β-conglycinin (7S) and glycinin (11S), respectively. 7S is composed of α′, α, and β subunits with molecular weights of 71 kDa, 67 kDa, and 50 kDa, respectively. 11S consists of six acidic peptides (molecular weights of 35–40 kDa) and six alkaline peptides (molecular weights of 20 kDa), each of which forms an AB subunit (molecular weights of 50 kDa) connected by disulfide bonds [23]. Figure 6 shows the molecular weight distribution of high-moisture extrusion of soybean proteins with different oil contents. The band intensity was significantly higher with the soybean oil groups than with the coconut oil groups, suggesting that the solubility of protein was relatively high in the presence of soybean oil. As the contents of soybean oil increased, the color of upper bands became darker while the color of lower bands became lighter (Figure 6a). As the contents of coconut oil increased, the color of upper bands became darker while the color of lower bands first became lighter and then darker. Based on densitometry analyses of the bands, the intensity of the texturized protein with soybean oil (Table 2) showed a significant increase at the 7S protein subunit region (α′, α, and β), a decrease at the 11S protein subunit region (A and B), and the 7S/11S ratio increased. In the texturized protein containing coconut oil, the intensity first decreased and then increased at the 7S protein subunit region and decreased at the 11S protein subunit region, and the 7S/11S ratio first decreased and then increased. This indicates that unsaturated fatty acids induced the aggregation of high-molecular-weight subunits, while saturated fatty acids induced the aggregation of medium- and low-molecular-weight subunits, thus enhancing the proportion of 11S protein subunits with a high content of sulfur-containing amino acids. Moreover, the fiber degree of the protein extrudates using coconut oil also was higher than those using soybean oil, suggesting that some sulfhydryl groups might undergo oxidation to form a disulfide bond during the extrusion of oil/protein composites, promoting cross-linking within the system, which enhanced the molecular weight of the protein [5].

### 3.6. Secondary Structure

**Figure 7:**
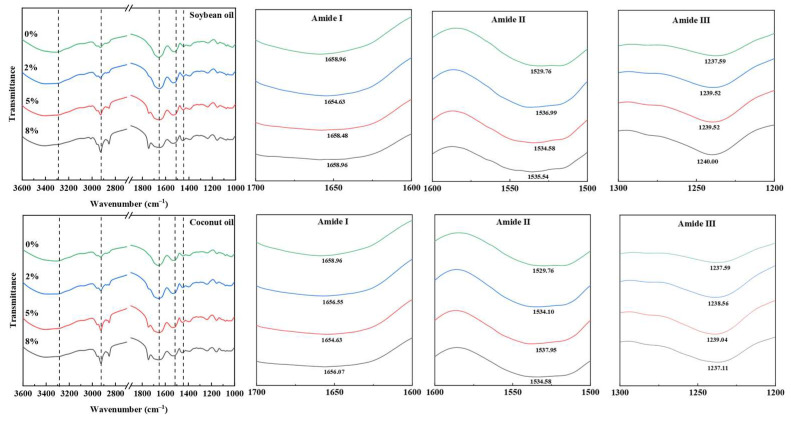
Effect of plant oil on the secondary structure of the texturized protein.

The FTIR of protein extrudates containing the two different plant oils is shown in Figure 7. The amide bands exhibited no significant shifts with the oil addition. An ester peak appeared at 1744 cm^−1^, and the highest band intensity was near 1658 cm^−1^, indicating that *β*-sheet and *β*-turn dominated the amide I band of the texturized proteins [24]. 

The secondary structure of protein extrudates is shown in Table 3, and the secondary structure content of protein extrudates was significantly altered by the addition of oils. Typically, the *α*-helix and *β*-sheet of proteins are located within the inner region of the polypeptide chains and can construct the rigid and stretchy skeleton of a protein by forming hydrogen bonds. The conformational stability of the β-turn and random coil was less strong and correlated with the flexibility of the protein molecules [25]. With the increase in oil concentration, there were no significant changes in the content of *α*-helix and random coil in protein extrudates, but the ratio of *α*-helix/*β*-sheet decreased, suggesting that the protein flexibility increased with the addition of oils. As the concentration of soybean oil increased, the content of *β*-sheet increased and then decreased, while the content of *β*-turn decreased and then increased, indicating a partial transformation of *β*-turn to *β*-sheet. When the content of soybean oil was 5%, the ratio of the *β*-sheet was the highest. With an increased coconut oil concentration, the content of *β*-sheet increased, while the content of *β*-turn decreased. The results showed that the *β*-sheet was associated with the intramolecular hydrogen bond interactions within the protein chain, which helped in maintaining the rigidity of high-moisture extrudates. This suggested that adding appropriate plant oils could increase the content of *β*-sheet of protein extrudates, enhancing the hydrogen bond between protein molecules. However, excessive oil could lead to protein structure unfolding, decreasing the stability of secondary structural conformation, which adversely affected the textural and sensory properties of the protein extrudates [26].

### 3.7. Protein Solubility and Chemical Cross-Linking

**Figure 8:**
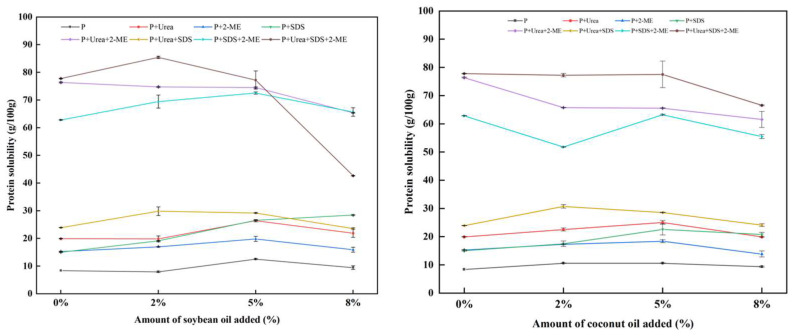
Effect of plant oil on the solubility of the texturized protein in different solvents.

The intermolecular force of extrudates was determined based on the varying protein solubility in the extraction solutions. The protein solubility of the extrudates in the eight extraction solutions is shown in Figure 8. In the two-component solvents, as the oil content increased, the protein solubility in P + urea and P + 2-ME showed a trend of first rising and then declining, and the solubility decreased when the oil content was above 5%. After adding oil, the solubility of protein in P + SDS was higher than that of the control group. This verified that adding a small quantity of oil promoted the formation of an intermolecular hydrogen bond and disulfide bond, and the introduction of long-chain fatty acids enhanced the hydrophobic interactions of the system. Our results aligned with the study carried out by Wang et al. [27], who found that hydrophobic interactions, hydrogen bonds, and disulfide bonds play crucial roles in preserving the fibrous structure of extrudates.

Table 4 shows the chemical bond content of texturized protein products. As the oil content increased, the content of the hydrogen bond and disulfide bond in the protein extracted first increased and then decreased, and the hydrophobic interactions increased. Overall, the addition of oils enhanced the interactions of the chemical bond. In the extracted protein, the ratio of the hydrogen bond and hydrophobic interactions was the smallest, while the interactions between hydrophobic interactions and the disulfide bond were moderate, and the ratio of the interactions between the hydrogen bond and disulfide bond was the biggest. This indicated that the hydrogen bond and disulfide bond were crucial for preserving the protein structure during extrusion. When the oil content was 5%, the content of the hydrogen bond and disulfide bond in the coconut oil groups is much higher than that in the soybean oil groups. This result suggested that plant oils rich in saturated fatty acids might facilitate chemical cross-linking between protein molecules, thus forming more compact and homogeneous fibrous structures [28].

### 3.8. Thermal Properties

**Figure 9:**
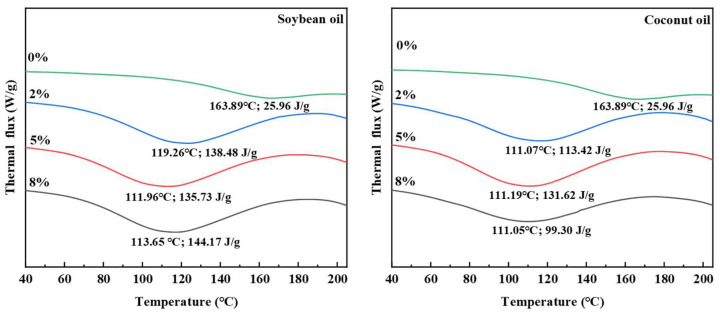
Effect of plant oil on the thermal properties of the texturized protein.

DSC is a commonly used method for studying protein thermal denaturation and thermodynamics [29,30]. The effect of plant oils on the thermal properties and order structure of texturized proteins during extrusion was analyzed through DSC. As shown in Figure 9 and Table 5, the addition of oils reduced the peak temperature (T_p_) but significantly increased the ΔH of the texturized proteins. With the increase in the content of oil, the ΔH of the soybean oil groups first decreased and then increased, the ΔH of the coconut oil groups initially increased and then decreased, and the ΔH of the plant oil groups was significantly higher than that of the control group. In addition, with the increase in the content of oil, the T_p_ of the soybean oil groups and coconut oil groups remained relatively constant. The reason for these results could be that adding oils decreased the degree of protein denaturation during extrusion, inhibiting cross-linking and bond formation, thus lowering the energy required for protein structure disruption [31]. Furthermore, the notable increase in ΔH suggests that the oil addition enhanced the order of protein structure and reduced the texture characteristics of the texturized protein products.

## 4. Conclusions

This study evaluated the effect of plant oils on the physicochemical characteristics of soy protein–wheat protein extrusion. The addition of plant oils enhanced the interaction forces (hydrogen bond and disulfide bond) between proteins, which promoted the occurrence of protein cross-linking and aggregation behavior during the cooling process. The coconut oil groups outperformed the soybean oil groups in terms of morphology, color, and fibrous structure. In the extruder barrel, the plant oils, especially coconut oil, promoted the oxidation of sulfhydryl groups to form disulfide bonds, enhancing cross-linking within the system, which enhanced the molecular weight of the protein. The coalescence of soybean oil, rich in unsaturated fatty acids, hindered the formation of fiber structures, while coconut oil dispersed uniformly in the protein matrix, increased disulfide bond formation, and promoted the formation of compact and homogeneous fiber structures. The addition of plant oils reduced the energy required for protein structure disruption during extrusion and promoted the conversion of *β*-turn to *β*-sheet coil conformations during high-moisture extrusion processing, thus enhancing the ordered structure of the protein. Therefore, the results suggested that the application of lipids composed of more fatty acids with lower unsaturation degree, such as coconut oil, would be favorable for generating protein fibrous structures. This study could provide a better understanding for the regulation and development of different plant oils’ impact on extrudate quality in high-moisture extrusion processing on a molecular level.

## Figures and Tables

**Table 1:** Sensory scoring of texturized protein products.

Attribute (Weight)	Quality Description and Scoring Criteria			
	1–3 Points	4–6 Points	7–9 Points	10 Points
Appearance (0.1)	Unshaped, loose, and rough surface	Minimally shaped, surface with fractures and granules	Adequately shaped, smooth surface, moderate texture	Perfectly shaped, smooth surface, uniform texture
Color (0.1)	Abnormal color	Dark and burnt color	Uniform color, slightly shiny	Uniform color, shiny
Texture (0.4)	Without fibrous structure	Poor fibrous structure, nonuniform arrangement, layered structure	Clear fibrous structure, relatively uniform arrangement, without layered structure	Clear fibrous structure, uniform arrangement, without layered structure
Flavor (0.1)	Poor flavor, heavy soybean odor	Moderate flavor, slight unpleasant odor	Good flavor, moderate meat aroma and taste, very slight unpleasant odor	Abundant meat aroma and taste, without unpleasant odor
Mouthfeel (0.3)	Without springiness, rough mouthfeel, low chewiness	Moderate springiness, hard mouthfeel, low chewiness	High springiness, good mouthfeel, moderate chewiness	High springiness, good mouthfeel, high chewiness

**Table 2:** Effects of plant oil on the molecular weight distribution of the texturized protein, presented as percentage of protein subunits (%).

Plant Oil Content	76 kDa	70 kDa	53 kDa	7S	41 kDa	35 kDa	21.4 kDa	17.8 kDa	11S	7S/11S
Control	4.52 ± 0.10 ^d^	3.67 ± 0.12 ^c^	3.48 ± 0.15 ^cd^	11.66 ± 0.13 ^d^	16.28 ± 0.69 ^a^	51.55 ± 0.29 ^bc^	1.98 ± 0.77 ^a^	18.54 ± 1.63 ^a^	88.34 ± 0.13 ^b^	0.13 ± 0.00 ^d^
Soybean oil (2%)	10.75 ± 0.13 ^a^	8.96 ± 0.39 ^b^	5.14 ± 0.90 ^ab^	24.85 ± 1.42 ^ab^	8.44 ± 2.37 ^d^	44.97 ± 3.43 ^d^	0.79 ± 0.55 ^a^	20.96 ± 0.90 ^a^	75.16 ± 1.42 ^de^	0.34 ± 0.03 ^b^
Soybean oil (5%)	8.84 ± 0.09 ^b^	8.25 ± 0.53 ^b^	5.92 ± 0.16 ^ab^	23.00 ± 0.59 ^bc^	9.62 ± 0.04 ^cd^	46.53 ± 1.52 ^cd^	1.35 ± 0.15 ^a^	19.50 ± 1.11 ^a^	77.00 ± 0.59 ^cd^	0.30 ± 0.01 ^b^
Soybean oil (8%)	9.94 ± 0.03 ^a^	12.51 ± 0.36 ^a^	6.45 ± 0.13 ^a^	28.89 ± 0.21 ^a^	8.53 ± 1.18 ^d^	42.24 ± 1.50 ^d^	1.03 ± 0.22 ^a^	19.31 ± 2.67 ^a^	71.11 ± 0.21 ^e^	0.41 ± 0.00 ^a^
Coconut oil (2%)	2.51 ± 0.44 ^e^	2.23 ± 0.42 ^c^	2.31 ± 0.56 ^d^	7.05 ± 1.42 ^e^	15.09 ± 1.31 ^ab^	54.37 ± 0.29 ^ab^	1.89 ± 1.33 ^a^	21.61 ± 1.15 ^a^	92.96 ± 1.42 ^a^	0.08 ± 0.02 ^d^
Coconut oil (5%)	3.21 ± 0.68 ^e^	3.67 ± 2.04 ^c^	2.15 ± 0.29 ^d^	9.03 ± 2.43 ^de^	13.12 ± 0.15 ^abc^	59.44 ± 2.09 ^a^	1.14 ± 0.64 ^a^	17.28 ± 5.01 ^a^	90.97 ± 2.43 ^ab^	0.10 ± 0.03 ^d^
Coconut oil (8%)	7.12 ± 0.15 ^c^	7.74 ± 0.76 ^b^	4.77 ± 0.06 ^bc^	19.63 ± 0.67 ^c^	12.00 ± 0.21 ^bcd^	42.78 ± 1.59 ^d^	1.45 ± 0.21 ^a^	24.15 ± 0.50 ^a^	80.37 ± 0.68 ^c^	0.24 ± 0.01 ^c^

^a–e^ There are significant differences between different letters.

**Table 3:** Effects of plant oil content on the relative percentage of protein secondary structure of the extrudates.

Plant Oil Content	*β*-Sheet (%)	Random Coil (%)	*α*-Helix (%)	*β*-Turn (%)
Control	27.99 ± 1.23 ^d^	20.65 ± 0.41 ^d^	21.94 ± 0.06 ^a^	29.41 ± 0.88 ^a^
Soybean oil (2%)	30.69 ± 0.25 ^bc^	21.28 ± 0.10 ^c^	21.41 ± 0.18 ^a^	26.62 ± 0.02 ^b^
Soybean oil (5%)	31.60 ± 0.18 ^ab^	21.82 ± 0.16 ^b^	20.14 ± 0.34 ^b^	26.44 ± 0.68 ^b^
Soybean oil (8%)	29.56 ± 1.31 ^cd^	22.76 ± 0.15 ^a^	18.97 ± 0.07 ^c^	28.71 ± 1.09 ^a^
Coconut oil (2%)	32.06 ± 0.42 ^ab^	21.30 ± 0.10 ^c^	20.53 ± 0.26 ^b^	26.12 ± 0.05 ^b^
Coconut oil (5%)	32.37 ± 0.95 ^ab^	21.92 ± 0.09 ^b^	20.44 ± 0.75 ^b^	25.27 ± 0.10 ^bc^
Coconut oil (8%)	32.90 ± 0.11 ^a^	22.17 ± 0.48 ^b^	20.53 ± 0.15 ^b^	24.40 ± 0.04 ^c^

^a–d^ There are significant differences between different letters.

**Table 4:** Effects of plant oil on chemical bond content of the texturized protein products, presented as protein solubility (g/100 g).

Plant Oil Content	Hydrogen Bond (A)	Disulfide Bond (B)	Hydrophobic Interactions (C)	A + B	A + C	C + B	A + B + C
Control	11.55 ± 0.12 ^bc^	6.90 ± 0.12 ^b^	6.65 ± 0.24 ^c^	49.54 ± 0.24 ^a^	−2.66 ± 0.01 ^b^	40.90 ± 0.24 ^a^	−43.47 ± 0.35 ^b^
Soybean oil (2%)	11.93 ± 1.87 ^bc^	9.04 ± 0.22 ^a^	11.23 ± 0.22 ^b^	45.84 ± 1.98 ^a^	−1.25 ± 0.44 ^ab^	41.24 ± 3.20 ^a^	−40.54 ± 3.09 ^b^
Soybean oil (5%)	13.87 ± 0.35 ^ab^	7.31 ± 1.51 ^ab^	14.04 ± 0.12 ^b^	40.80 ± 0.58 ^b^	−11.25 ± 0.58 ^d^	38.67 ± 1.97 ^a^	−38.75 ± 3.72 ^b^
Soybean oil (8%)	12.45 ± 1.13 ^abc^	4.47 ± 1.26 ^c^	19.01 ± 0.72 ^a^	36.99 ± 2.47 ^bc^	−17.33 ± 1.03 ^e^	30.73 ± 1.65 ^b^	−55.12 ± 0.31 ^c^
Coconut oil (2%)	11.25 ± 0.59 ^c^	6.33 ± 0.01 ^bc^	6.50 ± 1.18 ^c^	34.41 ± 0.59 ^cd^	1.25 ± 1.06 ^a^	25.99 ± 1.18 ^c^	−22.91 ± 0.12 ^a^
Coconut oil (5%)	14.46 ± 0.85 ^a^	7.79 ± 0.85 ^ab^	11.98 ± 2.66 ^b^	32.69 ± 0.24 ^d^	−8.47 ± 3.75 ^cd^	32.86 ± 1.69 ^b^	−24.39 ± 3.75 ^a^
Coconut oil (8%)	10.54 ± 0.01 ^c^	6.48 ± 0.31 ^bc^	11.44 ± 1.26 ^b^	37.17 ± 2.53 ^bc^	−7.24 ± 0.38 ^c^	30.20 ± 0.76 ^bc^	−29.39 ± 4.17 ^a^

^a–e^ There are significant differences between different letters.

**Table 5:** Effects of plant oil on the thermal properties of texturized protein.

Plant Oil Content	T_p_ (°C)	ΔH (J/g)
Control	163.89 ± 0.62 ^a^	25.96 ± 0.65 ^e^
Soybean oil (2%)	119.26 ± 3.75 ^b^	138.48 ± 6.70 ^ab^
Soybean oil (5%)	111.96 ± 0.21 ^c^	135.73 ± 3.05 ^b^
Soybean oil (8%)	113.65 ± 3.07 ^bc^	144.17 ± 3.04 ^a^
Coconut oil (2%)	111.07 ± 5.29 ^c^	113.42 ± 1.04 ^c^
Coconut oil (5%)	111.19 ± 1.67 ^c^	131.62 ± 3.01 ^b^
Coconut oil (8%)	111.05 ± 3.07 ^c^	99.30 ± 0.97 ^d^

^a–e^ There are significant differences between different letters.

## Data Availability

The data presented in this study are available on request from the corresponding author due to ongoing research using a part of the data.

## References

[1] Mattick C., Allenby B. (2013). The future of meat. Issues Sci. Technol..

[2] Ou M.J., Lou J.M., Lao L.F., Guo Y.X., Pan D.D., Yang H., Wu Z. (2023). Plant-based meat analogue of soy proteins by the multi-strain solid-state mixing fermentation. Food Chem..

[3] Dai H.H., An H.Z., Ma Y.X., Guo Y.T., Du Y., Zhu X.Q., Luo Q. (2023). Effects of lysine on the physiochemical properties of plant-protein high-moisture extrudates. LWT–Food Sci. Technol..

[4] Murillo J.S., Osen R., Hiermaier S., Ganzenmüller G. (2019). Towards understanding the mechanism of fibrous texture formation during high-moisture extrusion of meat substitutes. J. Food Eng..

[5] Zhang J.C., Liu L., Jiang Y.R., Shah F., Xu Y.J., Wang Q. (2020). High-moisture extrusion of peanut protein-/carrageenan/sodium alginate/wheat starch mixtures: Effect of different exogenous polysaccharides on the process forming a fibrous structure. Food Hydrocoll..

[6] Zasypkin D.V., Lee T.C. (1998). Extrusion of soybean and wheat flour as affected by moisture content. J. Food Sci..

[7] Lin S., Huff H.E., Hsieh F. (2002). Extrusion process parameters, sensory characteristics, and structural properties of a high moisture soy protein meat analog. J. Food Sci..

[8] Li R.L., Xue H., Gao B.H., Liu H.L., Han T.F., Hu X.B., Tu Y.G., Zhao Y. (2002). Study on the enhancement effect and mechanism of heat-induced gel strength of duck egg white by emulsified lipids. LWT–Food Sci. Technol..

[9] Devi A., Khatkar B.S. (2018). Effects of fatty acids composition and microstructure properties of fats and oils on textural properties of dough and cookie quality. J. Food Sci. Technol..

[10] Kendler C., Duchardt A., Karbstein H.P., Emin M.A. (2021). Effect of oil content and oil addition point on the extrusion processing of wheat gluten-based meat analogues. Foods.

[11] Guo X.N., Gao F., Zhu K.X. (2020). Effect of fresh egg white addition on the quality characteristics and protein aggregation of oat noodles. Food Chem..

[12] Xiao Z.G., Li H., Wang Z., Tian J.J., Zhu W.G., Jin Z.Y., He D., Wang N., Zhu M.P. (2022). Extrusion preparation and quality characteristics of high moisture textured compound protein. J. Chin. Cereals Oils Assoc..

[13] Chen Q.L., Zhang J.C., Zhang Y.J., Wang Q. (2022). Effect of fatty acid saturation degree on the rheological properties of pea protein and its high-moisture extruded product quality. Food Chem..

[14] Chen F.L., Wei Y.M., Zhang B. (2011). Chemical cross-linking and molecular aggregation of soybean protein during extrusion cooking at low and high moisture content. LWT–Food Sci. Technol..

[15] Grabowska K.J., Tekidou S., Boom R.M., van der Goot A.J. (2014). Shear structuring as a new method to make anisotropic structures from soy–gluten blends. Food Res. Int..

[16] Chen Y., Liang Y., Jia F., Chen D., Zhang X., Wang Q., Wang J.S. (2021). Effect of extrusion temperature on the protein aggregation of wheat gluten with the addition of peanut oil during extrusion. Int. J. Biol. Macromol..

[17] Wang H., Wang R.C., Zhang L.T., Zhang W., Bakalis S., Li Y., Lametsch R. (2023). Physicochemical properties, texture, and in vitro protein digestibility in high-moisture extrudate with different oil/water ratio. Food Res. Int..

[18] Afizah M.N., Rizvi S.S. (2014). Functional properties of whey protein concentrate texturized at acidic pH: Effect of extrusion temperature. LWT–Food Sci. Technol..

[19] Mozafarpour R., Koocheki A., Milani E., Varidi M. (2019). Extruded soy protein as a novel emulsifier: Structure, interfacial activity and emulsifying property. Food Hydrocoll..

[20] Chen Q.L., Zhang J.C., Zhang Y.J., Meng S., Wang Q. (2021). Rheological properties of pea protein isolate-amylose/amylopectin mixtures and the application in the high-moisture extruded meat substitutes. Food Hydrocoll..

[21] Mohammad A., Olcott H.S., Fraenkel-Conrat H. (1949). The reaction of proteins with acetaldehyde. Arch. Biochem..

[22] Liu K., Hsieh F.H. (2008). Protein–protein interactions during high-moisture extrusion for fibrous meat analogues and comparison of protein solubility methods using different solvent systems. J. Agric. Food Chem..

[23] Nishinari K., Fang Y., Guo S., Phillips G.O. (2014). Soy proteins: A review on composition, aggregation and emulsification. Food Hydrocoll..

[24] Sun F.W., Li B.L., Guo Y.A., Wang Y.C., Cheng T.F., Yang Q.Y., Liu J., Fan Z.J., Guo Z.W., Wang Z.J. (2022). Effects of ultrasonic pretreatment of soybean protein isolate on the binding efficiency, structural changes, and bioavailability of a protein-luteolin nanodelivery system. Ultrason. Sonochem..

[25] Zhou L.Y., Zhang Y., Zhao C.B., Lin H.J., Wang Z.J., Wu F. (2017). Structural and functional properties of rice bran protein oxidized by peroxyl radicals. Int. J. Food Prop..

[26] Guo Z.W., Teng F., Huang Z.X., Lv B., Lv X.Q., Babich O., Yu W.H., Li Y., Wang Z.J., Jiang L.Z. (2020). Effects of material characteristics on the structural characteristics and flavor substances retention of meat analogs. Food Hydrocoll..

[27] Beck S.M., Knoerzer K., Arcot J. (2017). Effect of low moisture extrusion on a pea protein isolate’s expansion, solubility, molecular weight distribution and secondary structure as determined by Fourier Transform Infrared Spectroscopy (FTIR). J. Food Eng..

[28] Wang K.Q., Li C., Wang B.Z., Yang W., Luo S.Z., Zhao Y.Y., Jiang S.T., Mu D.D., Zheng Z. (2017). Formation of macromolecules in wheat gluten/starch mixtures during twin-screw extrusion: Effect of different additives. J. Sci. Food Agric..

[29] Guo X.Y., Peng Z.Q., Zhang Y.W., Liu B., Cui Y.Q. (2015). The solubility and conformational characteristics of porcine myosin as affected by the presence of L-lysine and L-histidine. Food Chem..

[30] Zhao G.L., Liu Y., Zhao M.M., Ren J.Y., Yang B. (2011). Enzymatic hydrolysis and their effects on conformational and functional properties of peanut protein isolate. Food Chem..

[31] Chávez-Murillo C.E., Veyna-Torres J.I., Cavazos-Tamez L.M., de la Rosa-Millán J., Serna-Saldívar S.O. (2018). Physicochemical characteristics, ATR-FTIR molecular interactions and in vitro starch and protein digestion of thermally-treated whole pulse flours. Food Res. Int..

